# Does Size Matter? Scaling of CO_2_ Emissions and U.S. Urban Areas

**DOI:** 10.1371/journal.pone.0064727

**Published:** 2013-06-04

**Authors:** Michail Fragkias, José Lobo, Deborah Strumsky, Karen C. Seto

**Affiliations:** 1 Department of Economics, Boise State University, Boise, Idaho, United States of America; 2 School of Sustainability, Arizona State University, Tempe, Arizona, United States of America; 3 Department of Geography & Earth Sciences, University of North Carolina-Charlotte, Charlotte, North Carolina, United States of America; 4 Yale School of Forestry & Environmental Studies, Yale University, New Haven, Connecticut, United States of America; University of Florida, United States of America

## Abstract

Urban areas consume more than 66% of the world’s energy and generate more than 70% of global greenhouse gas emissions. With the world’s population expected to reach 10 billion by 2100, nearly 90% of whom will live in urban areas, a critical question for planetary sustainability is how the size of cities affects energy use and carbon dioxide (CO_2_) emissions. Are larger cities more energy and emissions efficient than smaller ones? Do larger cities exhibit gains from economies of scale with regard to emissions? Here we examine the relationship between city size and CO_2_ emissions for U.S. metropolitan areas using a production accounting allocation of emissions. We find that for the time period of 1999–2008, CO_2_ emissions scale proportionally with urban population size. Contrary to theoretical expectations, larger cities are not more emissions efficient than smaller ones.

## Introduction

Urbanization is a hallmark of the 21^st^ century, characterized by massive demographic shifts and large-scale rapid expansion of urban areas and the built environment [1]. Recent estimates show that 60–80% of final energy use globally is consumed by urban areas [2] and more than 70% of global greenhouse gas emissions are produced within urban areas [3]. The majority of future population growth for the remainder of this century will occur in urban areas [4]. The increase in global energy consumption, due to a rise in population and wealth will have significant effects on greenhouse gas emissions, human wellbeing, and sustainability [5]–[6].

It is a stylized fact that cities offer benefits from economies of scale. The concentration of people, large scale infrastructure and economic activity enable innovation and efficiencies [7]. Per capita urban energy consumption in industrialized countries is often lower than national averages [8]. Several studies show that compact and mixed urban land use coupled with co-located high residential and employment densities can reduce energy consumption and emissions through reducing vehicle miles traveled [9]–[10]. In this paper, we examine the relationship between population size of cities and carbon dioxide (CO_2_) emissions using data from the U.S. urban system.

One of the most salient characteristics of an urban area is it population size as it is both determinant and consequent of the socio-economic activity occurring within cities [11]. Urban population size has attracted significant attention across different disciplines as an indicator of the city and an explanandum of urban phenomena. A large body of literature in economics shows that larger urban agglomerations are more productive [7], [12] and more innovative [13]–[16]. The positive and strong relationship between urban size and productivity appears to be central characteristic of modern urban economies [17]. The importance of population size as a major factor in determining the intensity of socio-economic activity in urban areas has recently been emphasized by research that applies scaling analysis to a diverse spectrum of urban indicators [11], [18]–[19]. Scaling analysis, which has been a powerful tool across many scientific domains, represents how measurable aggregate characteristics respond to a change in the size of the system. Its analytical strength stems from the observation that this response is often a simple, regular, and systematic function over a wide range of sizes, indicating that there are underlying generic constraints at work on the system as it grows.

The population size of a city, as well as its spatial organization and structure can influence energy consumption. Energy is needed to both maintain existing infrastructure and to fuel economic activity while economic activity in turn affects energy demand [20], [21]. Calculations using a production-based accounting estimate that urban areas contribute approximately 30–40% of total anthropogenic greenhouse emissions - while, in contrast, a consumption-based accounting puts urban contributions at 60% of total, with a few wealthy cities contributing a majority of the emissions [8], [19], [22]. Data from world cities suggest that climate, technology, density and wealth are important determinants of energy use and CO_2_ emissions [23]. Past research has also shown that cities with larger populations present advantages over smaller cities in terms of their energy efficiency and CO_2_ emissions [24].

In this paper we examine the relationship between urban population size and urban CO_2_ emissions and ask the question: Are larger cities more emissions efficient than smaller ones? Furthermore, what is the relative importance of population size compared to other determinants of emissions discussed above? Given that urban populations will increase by 2–3 billion by the end of the 21^st^ century, understanding how urban size affects emissions can offer insight into how city size can be part of a larger regional or national strategy for reducing emissions. If larger cities are emissions efficient, national urban policy could encourage the development of large cities ceteris paribus - social, economic, and governance issues aside. Of course, urban and development policies would be constrained by other goals that cities–especially those in developing countries–are trying to achieve, including pollution abatement, poverty reduction, and industrialization, among others. Nonetheless, without fundamental scientific understanding of the relationship between urban population size and urban emissions, it is difficult for cities and national governments to prioritize sustainability and urbanization policies.

### The Importance of Scale for Urban CO_2_ Emissions

Scaling characterizes how a given systemic quantity of interest, *Y,* depends on the size of a system. A common feature of scaling is *scale invariance*, formalized as:
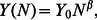
(1)where *Υ_0_* is a normalization constant and *β* is the scaling exponent, which can also be interpreted as an elasticity as usually defined in economics [25]. The significance of this “power law” relation becomes evident when we consider an arbitrary scale change by a factor *λ* from *N* to *λN*. This induces a change in *Y* from *Y(N)* to *Y(λN)* that can be expressed as



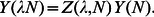
(2)This equation expresses the relation between *Y* for a system of size *N*, to *Y* for a system *λ* times larger. When the scale factor *Z* depends only on *λ*, i.e. 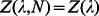, equation (2) can be solved *uniquely* to give the scale-invariant result of equation (1) with 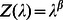. Scale-invariance implies that such a relationship – the ratio *Y(λN)/Y(N)* – is parameterized by a single dimensionless number *β*, usually referred to as the *scaling exponent*. The quantity *Y(λN)/Y(N)* is independent of the particular system size *N* but is dependent on the ratio between sizes *λ*. This behavior is what produces the linear relationship when logarithms are taken of both sides of equation (1), and the resulting straight-line on a log-log plot is the signature of a power law.

Recent research has pinpointed that cities can exhibit distinct types of scaling relationships across various urban phenomena or properties [11]. Sub-linear scaling (when the *β* exponents take a value of less than 1) parallels the allometric scaling laws observed in living organisms and represents the existence of economies of scale arising from an increase in efficiencies through the sharing of infrastructure; it is exhibited in electrical grids (through the length of electrical cables) and road systems (length of roads or amount of road surface) among other things. Super-linear scaling (when the *β* exponent is greater than 1) appears to be unique to social systems and is closely associated with the concept of network effects that lead to human ingenuity and creativity. Super-linear scaling has been identified in the number of new patents, inventors, R&D employment, total wages, etc. Linear scaling (when the *β* exponent is approximately equal to 1) signifies a proportional increase in urban phenomena/metrics with size.

The observation of scale invariance implies that the effects of increasing population size are general and can be observed by comparing any two cities, regardless of their size. If, for example, *Y* measures economic output, and two urban areas have population sizes of *N* and *λN*, respectively, scaling implies that the ratio of their outputs is a function of the proportion of their population sizes *λ*, but not of *N*. Scaling relations manifest an important empirical property: the phenomenon, repeats itself (albeit nontrivially) on different scales [26]. Such repetition points to possible underlying dynamical or stochastic processes generating and maintaining the same relationship among structural and functional variables over the range of the scale – typically many orders of magnitude [27]. A well-known example of a scaling relationship in the urban realm is “Zipf’s Law”, which states that a city’s population decreases in inverse proportion to its rank among other cities within the same urban system [28], [29].

Population size and energy consumption in cities have often been analyzed through the concept of “urban metabolism”. The concept of urban metabolism acknowledges that cities require a variety of inputs, among them energy, to maintain structure and remain functional [30]. Since its introduction in 1965, “urban metabolism” has become a widely used framework for understanding cities as both socio-economic and biophysical entities [31]–[36]. However, if CO_2_ emissions can be interpreted as an indirect measure of urban energy use, the concept of urban metabolism invites a comparison with the biological realm. One of the most celebrated relationships in biology is the scaling relationship between metabolic rate and organismic mass. “Kleiber’s law” states that for a vast array of organisms, metabolic rate scales to the ¾ power of the animal’s mass [37]–[40]. That is, larger animals consume more energy than smaller ones but the rate at which energy is used increases less than proportionally to the increase in body size. Larger organisms are therefore more energy efficient than smaller ones. The analogy implicit in the widespread use of the concept of “urban metabolism” lends itself to a question: are larger urban areas more efficient (e.g. β <1) than smaller ones with regards to CO_2_ emissions?

Before proceeding to a discussion of the data and a presentation of results, we briefly address the use of level vs. per capita measures when examining a scaling relationship between two variables - as captured by Equation (1), specifically the usefulness of a per capita measure of CO_2_ (such as CO_2_ emissions per urban inhabitant) as compared to a measure of total CO_2_ emissions for a population. When applied to urban metrics this presumes that urban characteristics scale linearly with city population size. If a scaling relationship exists between a variable *Y* and population, dividing *Y* by population introduces a nonlinearity into the per capita measure thereby reducing its accuracy [19], [41], [42]. Behind the choice of the most adequate dependent variable - total or per capita CO_2_ emissions - lies a choice as to how to analytically approach cities: as *extensive* systems with constant size-independent densities (per capita quantities) or as *non-extensive* systems for which densities are non-intensive and thus highly variable [43].

Cities show extreme spatial and individual heterogeneity: individuals, households and businesses differ markedly with respect to their attributes and performance. There is no such thing as a representative business or average person inside the city. Furthermore, many of the properties of the basic constituting elements of a city depend on the size of the entire system. CO_2_ emissions, as an extensive property, is accurately recorded in the aggregate but not in terms of the individual contributions. A scaling relationship is therefore a meaningful way of capturing how scale affects CO_2_ emissions.

## Materials and Methods

We use CO_2_ emissions data from Project Vulcan that quantifies U.S. fossil fuel carbon dioxide emissions at 10 km×10 km grid and at the scale of individual factories, power plants, roadways and neighborhoods on an hourly basis [44]. CO_2_ emissions quantification utilizes datasets such as air quality emissions reporting, census data, highway vehicle use reports, energy use statistics, power plants emissions compliance reports, and econometric data [44], [45]. Furthermore, Vulcan includes significant process-level detail, dividing the emissions into 9 economic sectors and 23 fuel types [45]. We utilize the Vulcan data that is available at the level of counties for the years 1999 to 2008.

The U.S. spatial units of analysis are the 366 Metropolitan Statistical Areas (MSAs) and the 576 Micropolitan Areas, which together constitute the 942 urban ‘core based statistical areas’ (CBSAs) of the United States. An MSA is defined as an “urbanized area” (densely settled areas with a population of at least 50,000) comprised of a central county together with adjacent outlying counties having a high degree of social and economic integration with the central county as measured through commuting flows. The geographical boundaries of MSAs can thus be identified as the outer boundaries of the set of counties that comprise them. A Micropolitan Area is similarly defined but the urbanized area has a population of less than 50,000 but greater than 10,000. Note that the county definition for urban areas experienced very little change over the decade for which the data on carbon emissions is available. In 2010, 83.7% and 10% of the U.S. population resided in MSAs and micropolitan areas respectively; 6.3% lived outside of MSAs and micropolitan statistical areas [46].

CBSA definitions are independent of municipal or State governmental jurisdictions or boundaries; MSAs and Micropolitan Areas constitute in effect unified labor markets. The range of population sizes exhibited by Metropolitan and Micropolitan Areas goes from Tallulah, Louisiana, with 12,113 inhabitants in 2010, to the New York metropolitan area with a population of almost nineteen million. These varied places provide their inhabitants with a social experience recognizable as “urban.” The U.S. Census – through its Office of Management and Budget (OMB) Bulletins – updates and revises delineations of metropolitan and micropolitan areas periodically. Our dataset thus includes all “urban” settlements of the U.S., which generate approximately 97% of the nation’s economic output, house about 94% of the country’s population and occupy less than 23% of its total land area.

We aggregate the total population of each county in the U.S into the MSA and micropolitan totals, using data from the Department of Commerce’s Bureau of Economic Analysis (BEA). We also aggregate the total amount of CO_2_ emissions (measured in millions of metric tones) allocated to each county by the Vulcan Project into MSA and Micropolitan Area totals based on the 2008 county delineations for metropolitan and micropolitan areas provided by the Census Bureau. We then construct a panel dataset for the period 1999–2008. Note that we aggregate all of the sources of CO_2_ emissions because we are interested in the energetic aspect of urban life and not simply on any one component–it could be that the compact spatial form of cities is associated with gains in energy efficiencies but that these gains are offset by the increased consumption facilitated by higher productivity levels induced by larger urban agglomerations.

Following our emphasis on scaling effects, we hypothesize that urban CO_2_ emissions are closely related to population size and that it scales according to a power-law relationship measured by.
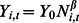
(3)where *Y* measures total CO_2_ emissions, *Y_0_* is a constant, *N* denotes population, *β* is the scaling exponent, and *i* and *t* index the urban area and year, respectively. This polynomial is a ubiquitous functional form commonly used in the natural and social sciences. Equation (3) acts as a baseline model and we let the data determine whether urban CO_2_ emissions are adequately modeled with a power-law relationship.

## Results

We use a decade of data for each urban area and across all urban areas to estimate a panel for Equation (1) using a generalized least squared framework which corrects for *AR(1)* autocorrelation within panels and cross-sectional correlation and heteroskedasticity across panels [47]. Our 930 cross-sectional observations across 10 years provide a total of 9,330 observations. Taking the logarithms of both sides of Eq. 3 and suppressing the panel (*i*,*t*) notation, our model yields the following result:
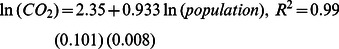
(4)


**Figure 1:**
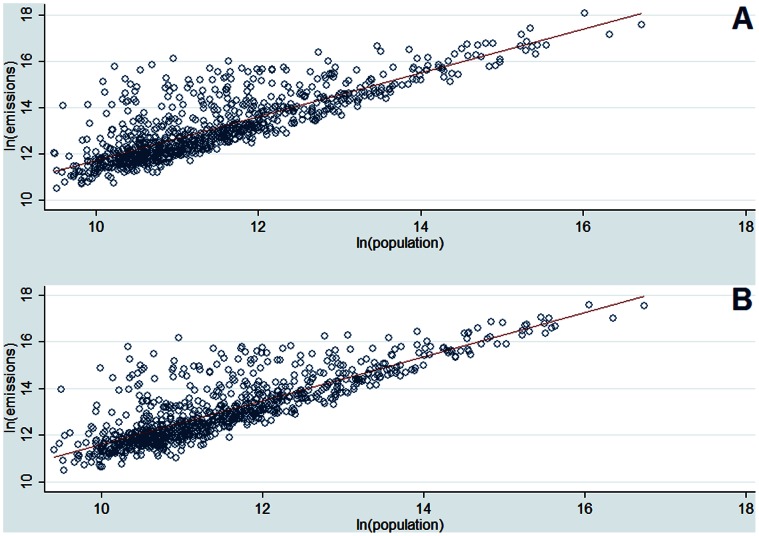
Cross-sectional log-log regressions for years (A) 1999 and (B) 2008.

The 95% confidence interval for the *ln(population)* coefficient in Eq. 4 is [.9164905,.9499573]. The coefficient is thus statistically different than 1. The scaling coefficient can be interpreted as elasticity, where a 1% increase in population size is associated with a nearly proportional increase in CO_2_ emissions of 0.93%. The value in parentheses is the heteroskedasticity-corrected standard error. Note that the same model and specification, run only for the subsample of MSAs for the 10 years (leading to a total of 3630 observations) yields a *ln(population)* coefficient of 0.90 and the same level of R^2^. We also conduct cross-sectional OLS estimations for each of the ten years for which data is available, done with a correction for heteroskedasticity; these regressions yield scaling coefficients in the order of 0.93–0.95 (a remarkable stability across time) and *R^2^* values ranging from 0.67–0.76. Using only the subsample of MSAs, the OLS estimations for each of the ten years, correcting for heteroskedasticity, yield scaling coefficients in the order of 0.91–0.92 and *R^2^* values ranging from 0.67–0.68. Figure 1 plots the cross-sectional regression results for the full sample and the two endpoint years in our dataset.

**Figure 2:**
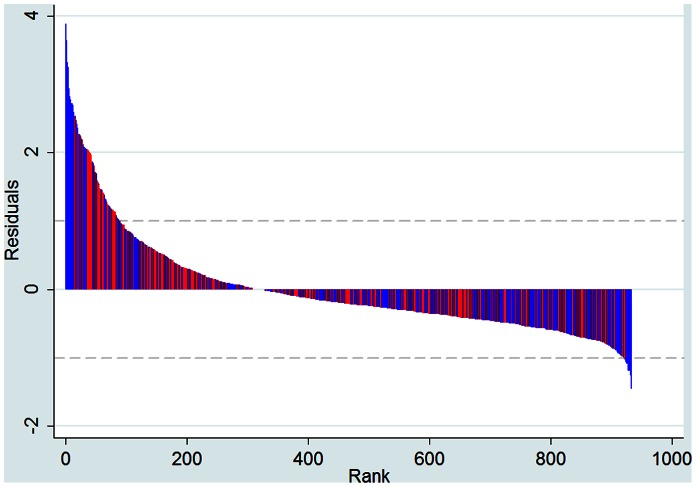
Ranking of residuals from the scaling regression for year 2008 (MSA observations in red; micropolitan area observations in blue).

Figure 2 plots the residuals from the full-sample cross-sectional regression for year 2008. Residuals range from a minimum value of −1.4 to a highest value of 3.9 but the vast majority range between [−1, 1]. Micropolitan areas produce the highest positive residuals and the highest negative residuals in our analysis, compared to MSAs.

Table 1 provides specific examples of the residuals ranking of the top 20 MSAS in the United States in year 2008. The biggest 20 MSAs in the U.S. span across a broad spectrum of the residuals ranking as shown in Figure 2. Cities like St. Louis and Minneapolis-St. Paul exhibit the highest positive deviations from the estimated scaling law in this subsample of the most populous MSAs. All MSAs that have positive residuals are considered to be underperforming in terms of CO_2_ emissions given their size. Cities like Los Angeles and Seattle exhibit the lowest negative deviations from the estimated scaling law in the subsample of MSAs. Cities with negative residuals are over-performing compared to the expectation based on their size.

**Table 1:** The 20 most populous MSAs in 2008 ranked by their deviation from the scaling law.

Top-20 MSAs (population) in 2008	Residual Rank	Deviation from scaling law
St. Louis, MO-IL	125	Positive
Minneapolis-St. Paul-Bloomington, MN-WI	158	Positive
Atlanta-Sandy Springs-Marietta, GA	195	Positive
Chicago-Joliet-Naperville, IL-IN-WI	209	Positive
Detroit-Warren-Livonia, MI	232	Positive
Houston-Sugar Land-Baytown, TX	236	Positive
San Francisco-Oakland-Fremont, CA	244	Positive
Tampa-St. Petersburg-Clearwater, FL	283	Positive
Baltimore-Towson, MD	307	Positive
Washington-Arlington-Alexandria, DC-VA-MD-WV	315	Positive
Phoenix-Mesa-Glendale, AZ	356	Negative
Boston-Cambridge-Quincy, MA-NH	432	Negative
Dallas-Fort Worth-Arlington, TX	475	Negative
Riverside-San Bernardino-Ontario, CA	485	Negative
Philadelphia-Camden-Wilmington, PA-NJ-DE-MD	533	Negative
San Diego-Carlsbad-San Marcos, CA	568	Negative
New York-Northern New Jersey-Long Island, NY-NJ-PA	664	Negative
Miami-Fort Lauderdale-Pompano Beach, FL	673	Negative
Seattle-Tacoma-Bellevue, WA	684	Negative
Los Angeles-Long Beach-Santa Ana, CA	778	Negative

Next, we enrich the relationship represented by Equation (4) with other important urban characteristics that may affect the energy consumption of urban areas: population density and residents’ wealth. Studies show that certain population density thresholds (that vary by location) are required to support public transport. Additionally, higher population densities, coupled with higher employment densities, also enable mixed land use, which in turn is critical for non-motor vehicle transport [10], [47]. Here, we use population density as an indicator of land use mix and urban form. Population density reflects urban form which in turn affects how much the mobility of urban residents depends on the use of vehicles. An urban area’s wealth is reflective of its economic composition and demographic characteristics, both of which may influence the intensity with which carbon-based fuels are used.

To control for the mediating effects of spatial form and wealth on the relationship between population size and urban energy use we add two independent variables to Equation (3), capturing the effects of urban wealth and population density. We define urban wealth as the *per capita personal income* (measured in current dollars). “Personal income” is the income received by individuals from all sources and is calculated as the sum of wage and salary disbursements, supplements to wages and salaries, personal transfers (such as social security payments), as well as proprietors', rental, dividend and interest income minus the contributions for government social insurance. “Per capita personal income” is obtained by dividing the total income accrued to the residents of an urban area by the area’s population. Data on urban PCPI is reported by the Department of Commerce’s Bureau of Economic Analysis (BEA).

We also create an urban population density measure that follows a population-weighted density definition [48], [49]. While a simple measure of density captures the ratio of urban population to total land area within the metropolitan boundaries, a population-weighted density measure resolves the problem of the non-uniform distribution of urban population within a city’s administrative boundaries. Thus, our density measure uses the proportion of total metropolitan population found within a county as weights, and provides a more accurate variable of urban density as experienced by the average urban inhabitant. While our intent is to use this density measure to control for the effects of land use mix and urban form on CO_2_ emissions it is important to note that the variable only imperfectly controls for the full range of potential urban form effects. Note that significant differences exist between the standard and the population-weighted density measures [46]. The New York MSA is almost twice as dense, while Phoenix is one and half times denser, using the population-weighted measure.

Including a measure for population density and per capita personal income as controls we obtain the following estimation results (Eq. 5) for a representative year (2008):
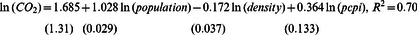
(5)


Robust standard errors are reported in the parentheses. The 95% confidence interval for the *ln(population)* coefficient in Eq. 5 is [.971, 1.084]; the coefficient is thus statistically indistinguishable from 1. This finding is replicated across all years in our study, with coefficients ranging from 1.02–1.03. While the effect of population is now linear (rather than near linear as discussed above), the results indicate that an increase in population density decreases CO_2_ emissions. In particular, in terms of elasticity, a 1% increase in our population-weighted density is associated with a 0.17% reduction in total CO_2_ emissions, ceteris paribus. Across all years in our study, the estimated coefficients for *ln(density)* range from −0.172 to −0.149. The effect of *density* is always statistically significant across the years in our study. Our findings suggest that while emissions drop with density, the benefits from the added density (such as trip savings or shortening) are overshadowed by the effects of the size of the metropolitan area.

Furthermore, our analysis shows that, controlling for urban population size and average density, in 2008, differences in wealth have a small positive effect on CO_2_ emissions – a 1% increase in *personal income* is associated with a 0.36% increase in total CO_2_ emissions, ceteris paribus. Across the years in our study, we find that this small positive effect of personal income is typically not statistically significant at the 1% level (it becomes statistically significant only in the latter years of our timeframe, post-2005, and the estimate coefficient ranges between 0.26 and 0.36). This finding in partially conflicting with the general consensus on the effect of wealth on CO_2_ emissions [23], [50]. Note that adding the density and wealth variables in the cross-sectional specification across all years does not improve the explanatory power of the models.

We also report the results utilizing the panel dataset and a generalized least squared framework which corrects for *AR(1)* autocorrelation within panels and cross-sectional correlation and heteroskedasticity across panels [47]; this approach though comes with a caveat: the personal income data is expressed in terms of nominal dollars (not real dollars), creating a challenge in the interpretation of the results from a panel regression (Eq. 6).
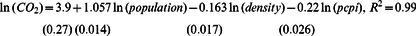
(6)


While the population and density explanatory variables yield the expected magnitude and sign, a 1% increase in personal income is now associated with a 0.22% decrease in expected total CO_2_ emissions, ceteris paribus.

## Discussion

Scaling is simply an emergent relationship between systemic size and emissions. Our results show that emissions in urban areas belong to a broader paradigm since every system needs to consume energy to maintain structure and order. The existence of approximate scaling phenomena for urban areas ― documented using a variety of socio-economic metrics ― is an indication that there are generic social mechanisms and properties of social systems at play across the entire urban system. Mechanisms such as networks and flows, nonlinearities and feedback loops integrate complex interactions among the individuals, households, firms, and institutions living, residing and operating in these spaces, leading to emergent phenomena such as scaling laws.

The near-linear relationship between population size and carbon emissions suggests that large urban areas in the U.S. are only slightly more emissions efficient than small ones. For each year in our sample, variation in population size across cities in the U.S. urban system explains approximately 70% of the variation of CO_2_ emissions with density and wealth not adding explanatory power to the models. This figure does not change when considering only MSAs – that is excluding settlements with populations between 10,000 and 50,000 people. This leaves a substantial proportion of the variation to be explained in the cross-sectional data by factors other than total population, density and wealth. Overall, stated in terms of CO_2_ emissions savings, cities in the US do not exhibit economies of scale on average (as defined by the elasticity concept we estimate in this paper) since they scale almost linearly. We suggest that this can only be claimed “on average” because we are not testing for scaling across different population types (e.g. we do not examine a potentially deviating scaling relationship arising from population specializing in distinct industrial sectors). That is, while more substantial economies of scale may be present when a city grows in terms of service sector or “creative” professionals, no economies may be present when the same cities adds manufacturing jobs. Our finding represents the average effect in the specific ten year evolution of the U.S. urban system. Controlling for variation in population density and wealth in cities does not alter our findings.

The intuitive interpretation of the linear scaling finding can be explored first through the analogy urban metabolism. Our finding creates a paradox when one considers that in nature, as organisms grow in size they become more efficient (see discussion on Kleiber’s Law above). A near-linear scaling in CO_2_ emissions, and thus only marginal gains in efficiency, casts some doubt on the hypothesis that urban systems function similarly to biological ones. While the analogy between urban metabolism and biological metabolism has been questioned before [36], our analysis provides further evidence that the analogy may have empirical limits. We now know that cities exhibit characteristics that make the natural organism analogy difficult, such as the urban phenomena that produce super-linear scaling [11]. Still, a theoretical possibility that energy use scales sub-linearly but CO_2_ emissions scale linearly; this would be the case if efficiencies in energy use where overshadowed by increased carbon intensiveness of the energy source mix that serves larger cities, the fossil fuel intensiveness of energy used in larger cities or the energy required to produce a unit of GDP in larger cities. Energy and emissions could scale differently because emissions are dependent on the type of energy used to generate final energy, the technology employed to use the energy, and the energy intensity of the economy [51].

We thus argue that an intuitive interpretation of the linear scaling finding requires an interpretation from economics, combined with an understanding of the nature of greenhouse gas emissions in the US. CO_2_ emissions depend significantly on the carbon intensity of the energy source and the drivers of demand for fossil fuels. Several hypotheses can be made on the basis of a decomposition of factors that drive demand for fossil fuels in localized markets. Expecting a pattern of increased savings in CO_2_ in larger urban agglomerations, a linear scaling of CO_2_ emissions may signify that larger urban areas are lagging in their capacity to curb demand for fossil fuels proportionally to smaller urban areas. Or, it may be the case that residents in larger urban areas are not incentivized structurally (through urban form) or economically (through energy prices) to demand lower proportions of fossil fuels in their energy mix. Furthermore, although large urban areas are more innovative than smaller ones, they may lack capacity in steering eco-innovations towards their local markets for fossil fuels. These important hypotheses remain untested and need to be addressed in future research.

Notwithstanding, our results have important energy policy implications for a rapidly urbanizing planet since they reveal the importance of urban scale/size relative to factors such as population density and wealth. The research shows that policymakers need to renew their attention on issues of distributions of city sizes within national urban systems; we show that size trumps the effects of all other variables (such as population density and wealth) in explaining variation in CO_2_ emissions. A focus on urban densities and wealth is still required, as these factors are critical for addressing various facets of global environmental change related to urban development. But as (new) world cities continue to grow, it is important that policymakers consider the CO_2_ emission effects of urban size and contrast it to the effects of urban form, building materials and transportation network structure. While we expect that scaling laws characterize the structure and order of urban systems globally, whether our specific U.S. results hold for all typologies of cities is beyond the scope of this study [52], [53].

The issues associated with emissions and energy accounting methods highlight the limitations of assuming cities as “closed systems”. The “closed system” perspective is in large part driven by the dominant conceptualization of a city through its narrow administrative boundaries – a definition of urban areas that drives data collection globally and dominates research practice surrounding urban phenomena. As we build our capacity to associate the increase of a city’s size to effects that occur far away from a city’s boundaries, we can overcome the data-specific challenge and adopt an “open system” perspective that could drastically alter our perspective on urban scaling. Through this new perspective, wealth, for example, may be found to be a more significant driver of total urban emissions; this is especially the case when considering emissions that occur in distal locations (or carbon sequestration capacity that is lost in distal places) but can be attributed to demand of goods and services that arises in specific urban areas [56]–[58].

Our “closed system” approach findings question the efficacy of using urban size as a climate change mitigation strategy. Our results show that, at least in the case of U.S. cities, there are no significant economies of scale with city size and CO_2_ emissions. Therefore, cities and policies must consider other mitigation strategies that have been shown to have greater impacts on emissions than population size. Furthermore, considering the policy relevance of these findings, we claim that limited economies of scale with respect to carbon emissions should be viewed in conjunction to the build-up of additional evidence on urban scaling. Any strategic decision on city growth considering sustainability will have to carefully weigh the implications of urban scale on a variety of urban metrics (including innovation, crime, environmental indicators, etc.). Our results contribute to the larger picture of scaling relationships present in urban systems: given that larger cities “speed up” the process of wealth creation and innovation [11] and do not offer significant economies of scale in CO_2_ emissions, a policy favoring larger city sizes may bring about carbon reductions primarily through technological advancements and eco-innovations.

## Supporting Information

Dataset S1 Basic panel dataset on CO_2_ emissions and population, CBSAs, 1999–2008 (co2_emission_panel.dta).
(DTA)

Dataset S2 Expanded panel dataset on CO_2_ emissions, population, population density and per capita personal income, CBSAs, 1999–2008 (mrg_pcpi_dens_1999–2008_long.dta).
(DTA)
